# Benefits of Dental Societies

**Published:** 1903-11-15

**Authors:** W. A. Barber


### BENEFITS OF DENTAL SOCIETIES.

BY W. A. BARBER, D.D.S.

The Dental Society, as an aid to growth, stands first and foremost. That this is the case, it seems to me, no argument is needed to prove. We have only to look about us for notable men in our profession to find prominent workers in dental societies. They may not have begun society or association work early in their professional lives, but sooner or later the need of the friction, drill, whip and spur of mind in contact with mind was felt so constantly and forcefully that they as naturally drifted to the society as the waters of enlarging rivers flow toward and into the ocean.

When once enrolled as members new growth began. They became conscious then, if not before, that others knew something. Perhaps they were surprised into belief that some others knew quite as much, or even a little more, than themselves. The elevation on which they stood, and fancied it a towering peak, began to seem like a moraine in a depressed valley; and to find associates they must scramble out of the plain and up toward real heights.

The coming in contact with men and women of high character and skill is of inestimable value.

When it is remembered that the occupation of the dentist necessarily confines him exclusively to his office during business hours, where he comes in contact with a limited number of people, it can readily be understood how such a life must shut off the broadening influence which contact with numerous people gives in most other occupations. We scarcely see any other individuals except the patients under our immediate care, and with these the important duty of serving each patient properly, combined with pain inflicted in so doing, makes our intercourse with them anything but pleasureable. And for many reasons it is especially important that the dentist should devise ways of having more social intercourse with his fellowman and especially with his fellow-practitioners.

Where this sociability exists it makes its impress on each individual. Dental societies, aside from their educational advantages, do much to give good feeling and to broaden the character of its members. Association stimulates ambition, developes the intellect and makes one more companionable. This is true of the professional as of the social side of life. Man is a social being; he has advanced under social conditions and there is in the professional as as well as in the mental and moral life profounder reasons for association as a means of education and professional growth than there are for mere material ends, and the time has come when we cannot afford even in a pecuniary sense to ignore associations.

There is a communication of mind with mind in which probably all who associate with one another participate, however unconsciously. There is not an individual, however humble, that does not have some influence, and when we put our minds in concord with others, when our hearts vibrate in harmony with them, how much we can do for the benefit of each other.

Association gives a man greater power for growth. The growing man learns something from everything he sees or comes in contact with. Nothing can touch him that does not teach him. The power to grow is fed by nothing so much as keeping the mind open to every possible suggestion from every possible source.

To surrender one's self to the education of life is to receive and give in the largest measure.

Progress is the eternal law of nature and in this progressive age, with the rapid strides our profession is taking, it is necessary for us to keep wide awake and use every energy at our command in order to keep in touch with it. It takes a better dentist to make a financial success now than it did ten years ago, and ten years hence the man who sticks to methods now in vogue will find himself more of a back number than the laggards of to-day.

The Dental Society largely contributes to the fraternal intercourse and good fellowship of its members, the interchanging of ideas through papers, discussions, and at our State meetings clinics increase our knowledge and is a well-recognized avenue through which the standard of our progress is brought to a higher plane to not only the members but to the world at large. Here it is that man of brains and genius professionally show their light, and what is their knowledge belongs to all of its members. It will be found that the leaders in any profession are leaders in association for the advancement of their chosen profession, be what it may.

The ministers have their associations, lawyers theirs, in fact all callings of life have their societies. He who scorns association necessarily places himself beyond the reach of progress; he will get into a rut and probabilities are he will stay there. We may be hiding away some golden thought that if brought forward into the light of controversy would prove to be of inestimable value.

The Dental Society has always exercised a helpful influence upon young and inexperienced practitioners, restraining characteristic impulses, cultivating and developing love of order and decent living. So that in a measure they become a necessity to localities, a means of protection, as well as a source of professional profit; if we find a town or a section with no society we may expect to find jealousies, envy, slander and all manner of bitterness, but all this has been known to clear away like smoke before the wind by the formation of a society.

Dentistry in America to-day leads the world, because American dentists have been teachers. Of what man in our ranks can it be said that he holds his knowledge, his skill, as men do secrets of trade? Is it not rather the fact that nothing brings greater joy to the clever and skillful among us than to have a message or method to give to his brother practitioners? Secrets and secret-mongers are despised among society men. Sixty years ago it was not an uncommon thing for those entering a dental office as assistants to be compelled to take an oath never to reveal the secrets imparted.

A little flight of time, not the span of a man's life, and quackery and commercial spirit give place to that broader spirit of humanity and liberality which must always be the guiding principle of that which claims a place among the learned professions.

Who but Horace H. Hayden was the man strong enough to set this examble for that which we now believe to be right?

Is not Carlyle again proved true in saying "The history of a nation is found in the biographes of her great men?"

That premature decay of the teeth is a result of civilization is an undeniable fact; and in those whose constitutions are depleted of the force the teeth are rarely good and they are only kept in fair working order by the great science of modern dentistry. Dentists are the barometers of civilization; their rise and prosperity is one of the most instructive facts in modern sociology. American dentists are the best because American teeth are the poorest in the world. Had the lives of pioneers, inventors and scientists in dentistry been measured by a century thrice told, their best efforts, singly exerted for that time, would have failed to produce results that by and through combined energy and society methods have been realized in a little more than a fourth of a century. Then does not the young dentist owe a debt that he is in honor bound to pay to the cause of science and progress?

Libraries, laboratories, colleges, post-graduate schools and curricula of technical courses that abound, but for those pioneers and for their society efforts, would not to-day have an existence.

Who knows? Who dares say that labor is lost, that a single thought shall perish?

Man is chiefly notable and distinguished not so much on account of what he is, as on account of what he is capable of doing, and in no profession do we find this truth so fully demonstrated. Life has but one meaning, and that is growth. Growth is the product of one force, and that is association. The growth of man means the unfolding and enlargement of his powers and capabilities. The hidden and higher processes by which his growth is accomplished lie on the side of mystery, but the human means by which his growth is fostered and promoted are of his own making and constitute his chief glory. In the constitution of the natural world there is everywhere an adaptation of this supreme end, "The growth of man," and no one force is as great a factor toward this end as association.

Let us catch an inspiration from the lives of the great who have gone before, and finally let us not forget "The assembling of ourselves together," where the good, old-fashioned brotherly love is trained and cultivated into professional spirit.

